# *ALDH2, ADH1B,* and *ADH1C* Genotypes in Asians: A Literature Review

**Published:** 2007

**Authors:** Mimy Y. Eng, Susan E. Luczak, Tamara L. Wall

**Keywords:** Alcohol dependence, ethanol metabolism, ethanol-to-acetaldehyde metabolism, alcohol dehydrogenase (ADH), aldehyde dehydrogenase (ALDH), acetaldehyde, ALDH2, ADH1B, ADH1C, risk factors, protective factors, genetic factors, ethnic groups, Asians, Chinese, Filipino, Indian, Japanese, Korean, Malaysian, Thai

## Abstract

Variants of three genes encoding alcohol-metabolizing enzymes, the aldehyde dehydrogenase gene ALDH2 and the alcohol dehydrogenase genes ADH1B and ADH1C, have been associated with reduced rates of alcohol dependence. The genotype prevalence of these genes varies in general samples of different Asian ethnic groups. The ALDH2*2 allele appears to be most prevalent in Chinese-American, Han Chinese and Taiwanese, Japanese, and Korean samples. Much lower rates have been reported in Thais, Filipinos, Indians, and Chinese and Taiwanese aborigines. ADH1B*2 is highly prevalent among Asians, with the exception of Indians. ADH1C*1 also is highly prevalent in Asians, but has only been examined in a few studies of Chinese and Korean samples.

People of Asian descent, as a whole, have lower rates of alcohol dependence compared with other ethnic groups (Grant et al. 2004). Within Asians, however, rates of alcohol dependence differ across ethnic subgroups. For example, relatively high rates of alcohol dependence have been found among Koreans and Korean Americans, whereas relatively low rates have been found in Chinese and Chinese Americans (Helzer et al. 1990; Luczak et al. 2004). Prevalence rates of alleles of genes encoding alcohol-metabolizing enzymes vary across Asian ethnicities (e.g., Goedde et al. 1992). This may in part account for some of the ethnic differences in rates of alcohol involvement. The purpose of this article is to review genotype^1^ prevalence rates of three genes, the aldehyde dehydrogenase gene *ALDH2* and the alcohol dehydrogenase genes *ADH1B* and *ADH1C*.^2^

These three genes code for isoenzymes that metabolize alcohol into acetaldehyde (*ADH1B* and *ADH1C*) and acetaldehyde into acetate (*ALDH2*). The common forms of these alleles are ADH1B*1, ADH1C*2, and ALDH2*1. The variant forms of the alleles (ADH1B*2, ADH1C*1, and ALDH2*2) are hypothesized to alter conversion rates during alcohol metabolism and lead to an excess buildup of acetaldehyde (see Eriksson 2001). The excess acetaldehyde is thought to lead to heightened responses to alcohol and thereby reduce heavy alcohol use, associated problems, and the development of alcohol use disorders (see Wall et al. 2005 for further details). A meta-analysis of 15 Asian (Chinese, Japanese, Korean, and Thai) studies with data from over 4,500 alcohol-dependent and control subjects collected between 1979 and 2004 found possession of one variant ALDH2*2 allele was associated with a fivefold reduction in alcohol is dependence and possession of two ALDH2*2 alleles was associated with a nine-fold reduction (Luczak et al. 2006).^3^ In Asians with no ALDH2*2 alleles, possession of one variant ADH1B*2 allele was associated with a four-fold reduction in alcohol dependence and possession of two ADH1B*2 alleles was associated with a five-fold reduction (Luczak et al. 2006). ADH1C*1 also has been related to protection against alcohol dependence, but this association has been attributed to the *ADH1C* gene being in close proximity to the *ADH1B* gene on the chromosome so that the genotypes are correlated (Osier et al. 1999).

Determining how frequently certain genotypes occur in different populations is useful for behavioral genetics research. It is important to establish the prevalence rates of these genotypes in various ethnic groups to determine their unique contribution to alcohol involvement within each ethnicity. To achieve this goal for Asian populations, an extensive literature review of studies determining the prevalence of the *ALDH2*, *ADH1B*, and *ADH1C* genotypes in various Asian ethnic groups was performed, as described in the following sections.

## Prevalence of *ALDH2*,*ADH1B*, and *ADH1C* Genotypes in Asian Populations

### Study Design

To identify studies eligible for this analysis, the authors of this article surveyed the Medline literature database using the National Library of Medicine’s PubMed (January 1966 to April 2006) online search engine. The search first was conducted using the keywords “(aldehyde dehydrogenase OR ALDH) AND Asian;” then, additional searches were conducted by replacing “Asian” with specific Asian ethnicities (i.e., Chinese, Filipino, Indian, Japanese, Korean, Malaysian, and Thai). The series of searches then was repeated using the keywords “(alcohol dehydrogenase OR ADH).” The retrieved abstracts were read to identify studies that reported prevalence rates of the various *ALDH2*, *ADH1B*, and *ADH1C* genotypes in general samples of the different ethnic groups. The studies thus identified were read in their entirety to assess whether they were appropriate for including in this analysis. Studies that reported only allele frequencies instead of genotypes, compared treatment samples with control groups, or selected samples based on specific alcohol-related medical conditions (e.g., cirrhosis or head and neck cancers) were excluded. All references cited in the appropriate articles also were reviewed to identify additional relevant publications.

Despite the stringent criteria for the selection of studies to be included, the following caveats should be noted:

- Some samples included in the analysis may not be entirely random because participants were screened for certain medical disorders (e.g., diabetes, heart conditions, and stroke) that have been related to alcohol in addition to other factors.
- Samples with allele distributions that do not meet Hardy-Weinberg equilibrium^4^ (which are marked in the table summarizing the results) should be viewed with caution because the genotype distribution in these studies is not consistent with the expected distribution for a general sample.

### Results of the Analysis

#### Distribution of ALDH2 Genotypes

The ALDH2*2 allele is thought to occur exclusively in Asians; however, its prevalence varies across Asian ethnicities (see Table 1). Five studies determined the *ALDH2* genotype in Han Chinese and Taiwanese people.^5^ In these studies, 20 to 47 percent of the participants were heterozygous and 1 to 8 percent were homozygous for ALDH2*2 (Goedde et al. 1992; Luo et al. 2001, 2005; Novoradovsky et al. 1995; Shen et al. 1997). Overall, approximately one-third of the Han Chinese possessed at least one ALDH2*2 allele. The prevalence of the ALDH2*2 allele was particularly high in one study of Han Taiwanese and two studies of Chinese Americans, with about half of these samples possessing at least one ALDH2*2 allele, including 7 to 8 percent who were homozygous for ALDH2*2 (Hendershot et al. 2005; Luczak et al. 2004; Novoradovsky et al. 1995). The large variation in prevalence rates found among Han Chinese and Taiwanese samples might be explained by the different geographic locations from which the samples were obtained. The sample with the highest prevalence was from Taiwan, where 55 percent of participants possessed at least one *ALDH2* allele (Novoradovsky et al. 1995). Conversely, the samples with the lowest prevalence were from central and northern China, where 22 percent of participants possessed at least one ALDH2*2 allele (Luo et al. 2001; Shen et al. 1997). For the studies with intermediate prevalence rates (i.e., 30 to 32 percent), the samples were from southwest China (Luo et al. 2005) or their location was not reported (Goedde et al. 1992).

The ALDH2*2 allele was less commonly found in aboriginal Chinese and Taiwanese samples (e.g., Ami, Atayal, Bunun, Elunchan, Mongolian, and Paiwan), with 2 to 12 percent of study participants being heterozygous and only 0.3 percent (i.e., 2 of 585 people analyzed) being homozygous for ALDH2*2 (Chen et al. 1997; Shen et al. 1997; Thomasson et al. 1994).

Data from 10 Japanese studies indicated that 41 to 52 percent of Japanese possessed at least one ALDH2*2 allele, including 1 to 8 percent who were homozygous for ALDH2*2 (Amamoto et al. 2002; Goedde et al. 1992; Higuchi et al. 1996; Saito et al. 2003; Sun et al. 1999; Takeshita and Morimoto 1999: Takeshita et al. 1994; Tanaka et al. 1997; Yamada et al. 2002; Yokoyama et al. 2005). Somewhat higher rates were reported in one small Japanese study (*N* = 15), in which 66 percent of the participants possessed at least one ALDH2*2 allele, including 13 percent who were homozygous for ALDH2*2 (Shibuya et al. 1989).

Five studies of Korean, Korean-American, and Korean-Chinese samples found that approximately one-third (29 to 37 percent) of Koreans had at least one ALDH2*2 allele, including 2 to 3 percent who were homozygous for ALDH2*2 (Goedde et al. 1992; Hendershot et al. 2005; Lee et al. 1997; Luczak et al. 2004; Shen et al. 1997). Finally, ALDH2*2 was much less common among other Asian ethnicities, including Filipinos, Indians, Malays, Siberian Yakuts, and Thais, than in Chinese, Japanese, and Korean samples, with 0 to 10 percent of study participants possessing at least one ALDH2*2 allele (Goedde et al. 1992; Novoradovsky et al. 1995). Taken together, all the studies reviewed here demonstrate great diversity among Asian ethnic groups in the prevalence of heterozy-gosity or homozygosity for ALDH2*2.

#### Distribution of ADH1B Genotypes

The ADH1B*2 allele was highly prevalent in Asian ethnic groups, particularly in northeast Asians (i.e., Chinese, Japanese, and Koreans) (see Table 1). Among the Han Chinese and Taiwanese and the Chinese Americans, 84 to 92 percent possessed at least one ADH1B*2 allele, including 40 to 60 percent who were homozygous for ADH1B*2 (Chao et al. 1987; Goedde et al. 1992; Lee et al. 1989; Luczak et al. 2004; Shen et al. 1997). Rates of having at least one ADH1B*2 allele were slightly lower in some Chinese and Taiwanese aborigine groups (e.g., 63 percent in Elunchan, 74 percent in Mongolian, and 78 percent in Ami) but were higher in others (e.g., 98 to 100 percent in Atayal, Bunun, and Paiwan) (Chen et al. 1997; Shen et al. 1997; Thomasson et al. 1994).

The ADH1B*2 allele also was commonly found in Japanese people. In 10 studies of Japanese, 81 to 100 percent of participants possessed at least one ADH1B*2 allele, including 34 to 71 percent who were homozygous for the allele (Goedde et al. 1992; Higuchi et al. 1996; Saito et al. 2003; Shibuya et al. 1989; Sun et al. 1999; Suzuki et al. 2004; Takeshita et al. 1996; Tanaka et al. 1997; Yamada et al. 2002; Yin et al. 1984). The results of one of the studies (Yin et al. 1984), in which ADH1B*2 prevalence rates were among the lowest for Japanese and Japanese Americans, however, must be viewed with caution because the distributions were not in Hardy-Weinberg equilibrium.

The prevalence of ADH1B*2 also was high in three Korean samples*,* with 88 to 96 percent of participants possessing at least one ADH1B*2 allele and 50 to 65 percent possessing two ADH1B*2 alleles (Goedde et al. 1992; Luczak et al. 2004; Shen et al. 1997). Among Filipinos and Malays, more than 80 percent of study participants carried at least one ADH1B*2 allele (Goedde et al. 1992) as well. Intermediate rates were found in Thais (54 percent), and ADH1B*2 was least common in Indians, where only 15 percent possessed at least one copy of the allele (Goedde et al. 1992).

#### Distribution of ADH1C Genotypes

*ADH1C* genotypes only have been examined in a few Chinese and Korean samples, but in these samples the ADH1C*1 allele was highly prevalent. In one study of Han Chinese, 97 percent of participants possessed at least one ADH1C*1 allele, including 83 percent who were homozygous (Shen et al. 1997). Comparably high proportions (97 to 100 percent) of seven Chinese aboriginal populations possessed at least one ADH1C*1 allele, although the rates of homozygosity for ADH1C*1 were more variable (59 to 99 percent) in these populations (Chen et al. 1997; Shen et al. 1997; Thomasson et al. 1994). Finally, the prevalence of ADH1C*1 in one Korean Chinese sample was similar to the rates reported in Chinese samples, with 99 percent of subjects possessing at least one ADH1C*1 allele, including 86 percent who were homozygous for the allele (Shen et al. 1997).

## Summary

This literature review highlights the fact that the prevalence of *ALDH2*, *ADH1B*, and *ADH1C* alleles vary greatly across Asian ethnic groups. For example, whereas approximately half of Chinese-American and Japanese samples and approximately one-third of Korean and Han Chinese and Taiwanese studied carry at least one ALDH2*2 allele, the prevalence of this allele is much lower (10 percent) in Thais, and almost no Filipinos, Indians, or Chinese and Taiwanese aborigines carry the allele, with the exception of Mongolians (12 percent). Similarly, the ADH1B*2 allele is found in 80 percent or more of Han Chinese and Taiwanese, Filipino, Japanese, Korean, and some Chinese and Taiwanese aborigine people but only in about 15 percent of Indians. Finally, the ADH1C*1 allele was found in almost all Chinese and Korean people studied, but it has not been analyzed yet in other Asian ethnic groups. Such summaries of general-sample prevalence rates are important for understanding risk and protective factors for alcohol use disorders because they facilitate comparisons of the contribution of these alcohol-metabolizing enzymes and their variants to alcohol-related behaviors within and across ethnic groups.

## Figures and Tables

**Table 1:** Genotypes for Genes Encoding Aldehyde Dehydrogenase *(ALDH2)* and Alcohol Dehydrogenase *(ADH1B* and *ADH1C)*

**Study Authors**	**Sample**	***ALDH2* Genotypes prevalence (%)**			***ADH1B* Genotypes prevalence (%)**			***ADH1C* Genotypes prevalence (%)**		

		*1/*1	*1/*2	*2/*2	*1/*1	*1/*2	*2/*2	*1/*1	*1/*2	*2/*2
**Han Chinese and Taiwanese**										
Chao et al. 1987	60 male and 11 female liver specimens				10	31	59			
Goedde et al. 1992	132 subjects*	70	29	1	8	48	44			
Lee et al. 1989	53 lung specimens				9	30	60			
Luo et al. 2001	50 subjects	78	20	2						
Luo et al. 2005	444 males and 204 females	68	28	4						
Novoradovsky et al. 1995	173 blood donors	45	47	8						
Shen et al. 1997^c^	100 male	78	20	2	16	44	40	83	14	3
**Total**		**66**	**30**	**4**	**11**	**40**	**49**	**83**	**14**	**3**
**Chinese American**										
Hendershot et al. 2005	110 male and 113 female college students	51	43	7						
Luczak et al. 2004	92 males and 98 females college students	48	44	8	8	33	58			
**Total**		**49**	**43**	**7**	**8**	**33**	**58**			
**Chinese and Taiwanese Aborigine**										
Chen et al. 1997										
Ami	46 subjects*	93	7	0	22	38	40	98	2	0
Atayal	67 subjects*	97	3	0	0	21	79	96	4	0
Bunun	118 subjects*	98	2	0	1	30	69	88	12	0
Paiwan	71 subjects*	95	5	0	0	31	69	99	1	0
Shen et al. 1997										
Elunchan^a^	68 males	93	6	1	37	54	9	59	38	3
Mongolian	66 males	88	12	0	26	44	30	73	26	2
Thomasson et al. 1994										
Atayal^a^	80 males and 80 females*	94	5	1	3	24	74	97	3	0
**Total**		**95**	**5**	**0**	**10**	**32**	**58**	**88**	**11**	**1**
**Filipino**										
Goedde et al. 1992	86 subjects*	99	1	0	19	40	40			
**Indian**										
Goedde et al. 1992^a,b^	179 subjects*	97	3	1	85	10	5			
**Japanese**										
Amamoto et al. 2002^a^	749 males and 1,286 females	48	45	7						
Goedde et al. 1992	53 subjects*	55	43	2	16	50	34			
Higuchi et al. 1996	230 male and 221 female hospital employees and relatives	59	35	6	7	35	58			
Saito et al. 2003	335 males	53	41	6	8	35	57			
Shibuya et al. 1989	15 males*	33	53	13	0	29	71			
Sun et al. 1999	643 male hospital and civil service employees	58	36	6	4	35	61			
Suzuki et al. 2004	1,126 males				5	34	61			
Takeshita & Morimoto 1999	389 males and 34 females medical students	54	40	5						
Takeshita et al. 1994	424 male and 100 females metal plant workers	57	37	7						
Takeshita et al. 1996	424 male and 100 females metal plant workers				6	33	60			
Tanaka et al. 1997^a^	189 males	51	48	1	5	38	57			
Yamada et al. 2002	855 male factory workers	58	36	6	4	36	60			
Yin et al. 1984^b^	97 liver samples				13	29	58			
Yokoyama et al. 2005	139 male and 112 female workers	59	33	8						
**Total**		**54**	**40**	**6**	**6**	**35**	**60**			
**Japanese American**										
Yin et al. 1984^b^	97 liver samples				19	34	47			
**Korean**										
Goedde et al. 1992	218 subjects*	72	27	2	4	31	65			
Lee et al. 1997	481 subjects	71	26	3						
**Total**		**71**	**26**	**3**	**4**	**31**	**65**			
**Korean American**										
Hendershot et al. 2005	97 male and 108 female college students	67	32	2						
Luczak et al. 2004	107 male and 107 female college students	66	31	3	10	36	53			
**Total**		**66**	**32**	**2**	**10**	**36**	**53**			
**Korean Chinese**										
Shen et al. 1997	105 males	63	34	3	11	38	50	86	13	1
**Malay**										
Goedde et al. 1992	73 subjects*	93	7	0	17	48	35			
**Thai**										
Goedde et al. 1992	111 subjects	90	10	0	46	41	13			
**Siberian Yakut**										
Novoradovsky et al. 1995	209 subjects	100	0	0						

a not in Hardy-Weinberg equilibrium for *ALDH2;*

b not in Hardy-Weinberg equilibrium for *ADH1B;*

c not in Hardy-Weinberg equilibrium for *ADH1C;*

df = 1, p < .05 for all.

* Sample size varies by gene analyzed.

## Footnotes

1 Every person possesses two copies of each allele; these two alleles make up the genotype.

2 *ADH1B* and *ADH1C* were formerly called *ADH2* and *ADH3,* respectively (for more information, see the accompanying article by Edenberg).

3 If a person has two copies of the same allele, the person is called homozygous for that allele; if the two copies are of different alleles, the person is called heterozygous.

4 Hardy-Weinberg equilibrium is the stable frequency distribution of genotypes, as measured by the proportion of the alleles that result as a consequence of random mating.

5 The Han are the main ethnic group found in the People’s Republic of China and Taiwan.
